# Acetylation of muscle creatine kinase negatively impacts high-energy phosphotransfer in heart failure

**DOI:** 10.1172/jci.insight.144301

**Published:** 2021-02-08

**Authors:** Matthew A. Walker, Juan Chavez, Outi Villet, Xiaoting Tang, Andrew Keller, James E. Bruce, Rong Tian

**Affiliations:** 1 Mitochondria and Metabolism Center, Department of Anesthesiology and Pain Medicine, and; 2 Department of Genome Sciences, University of Washington, Seattle, Washington 98109, USA.

**Keywords:** Cardiology, Heart failure

## Abstract

A hallmark of impaired myocardial energetics in failing hearts is the downregulation of the creatine kinase (CK) system. In heart failure patients and animal models, myocardial phosphocreatine content and the flux of the CK reaction are negatively correlated with the outcome of heart failure. While decreased CK activity is highly reproducible in failing hearts, the underlying mechanisms remains elusive. Here, we report an inverse relationship between the activity and acetylation of CK muscle form (CKM) in human and mouse failing hearts. Hyperacetylation of recombinant CKM disrupted MM homodimer formation and reduced enzymatic activity, which could be reversed by sirtuin 2 treatment. Mass spectrometry analysis identified multiple lysine residues on the MM dimer interface, which were hyperacetylated in the failing hearts. Molecular modeling of CK MM homodimer suggested that hyperacetylation prevented dimer formation through interfering salt bridges within and between the 2 monomers. Deacetylation by sirtuin 2 reduced acetylation of the critical lysine residues, improved dimer formation, and restored CKM activity from failing heart tissue. These findings reveal a potentially novel mechanism in the regulation of CK activity and provide a potential target for improving high-energy phosphoryl transfer in heart failure.

## Introduction

A hallmark of impaired energetics in the human failing heart is decreased creatine kinase (CK) activity (1–4). The CK reaction catalyzes the high-energy phosphoryl transfer between phosphocreatine (PCr) and ATP, thus providing an efficient energy shuttle mechanism for excitable tissues (5). Distinct genes encode M and B isoforms of CK that form stable homo and hetero dimers (MM, MB, BB) in cytosol (6). In their native state, cytosolic isozymes are stable dimers facilitating high-energy phosphate transfer from mitochondria to sites of ATP utilization. A third gene encodes mitochondrial isoform of the CK (MtCK) that forms both dimers and octamers, depending on pH and concentration. Changes in CK protein and gene expression levels have been mild and inconsistent in failing hearts (7, 8). However, through unknown mechanisms, decreased CK activity is highly reproducible in the failing hearts of all experimental and clinical studies, and it results in reductions in ATP delivery to the myofibrils by up to 71% (9). Localization of the muscle CK isoform (CKM) immediately proximal to the myosin ATP motor domain heads and to the ATP-dependent calcium pump (sarco-endoplasmic reticulum calcium-ATPase; SERCA) further illustrates this tight control for ADP/ATP concentrations needed for heart function (6, 10, 11). Downregulation of the CK system correlates with degree of cardiac dysfunction and/or the outcome of heart failure in animal models and patients (2, 4, 12, 13). Conversely, overexpression of CKM in mouse hearts preserves ATP production via the CK reaction and improves survival and contractile function in pressure overload–induced heart failure (8).

Recently, studies conducted in preclinical models of heart failure have shown relative increases in the abundance of nonhistone Nε-lysine acetylation of myocardial proteins (14–17). There is increasing evidence that protein hyperacetylation in human heart failure could be an important component driving the aforementioned energy deficit. In this study, we report that CK acetylation is negatively correlated with its activity in the failing hearts of mice and humans. We have further demonstrated that lysine acetylation in CKM impairs its enzymatic activity by disrupting the salt bridges required for dimer formation. These findings reveal a potentially novel mechanism in regulating high-energy phosphoryl transfer in heart failure, hence providing a potential target to intervene in myocardial energetic dysfunction in failing hearts.

## Results

### Lysine acetylation level on CK negatively correlates with its activity.

**Figure 1:**
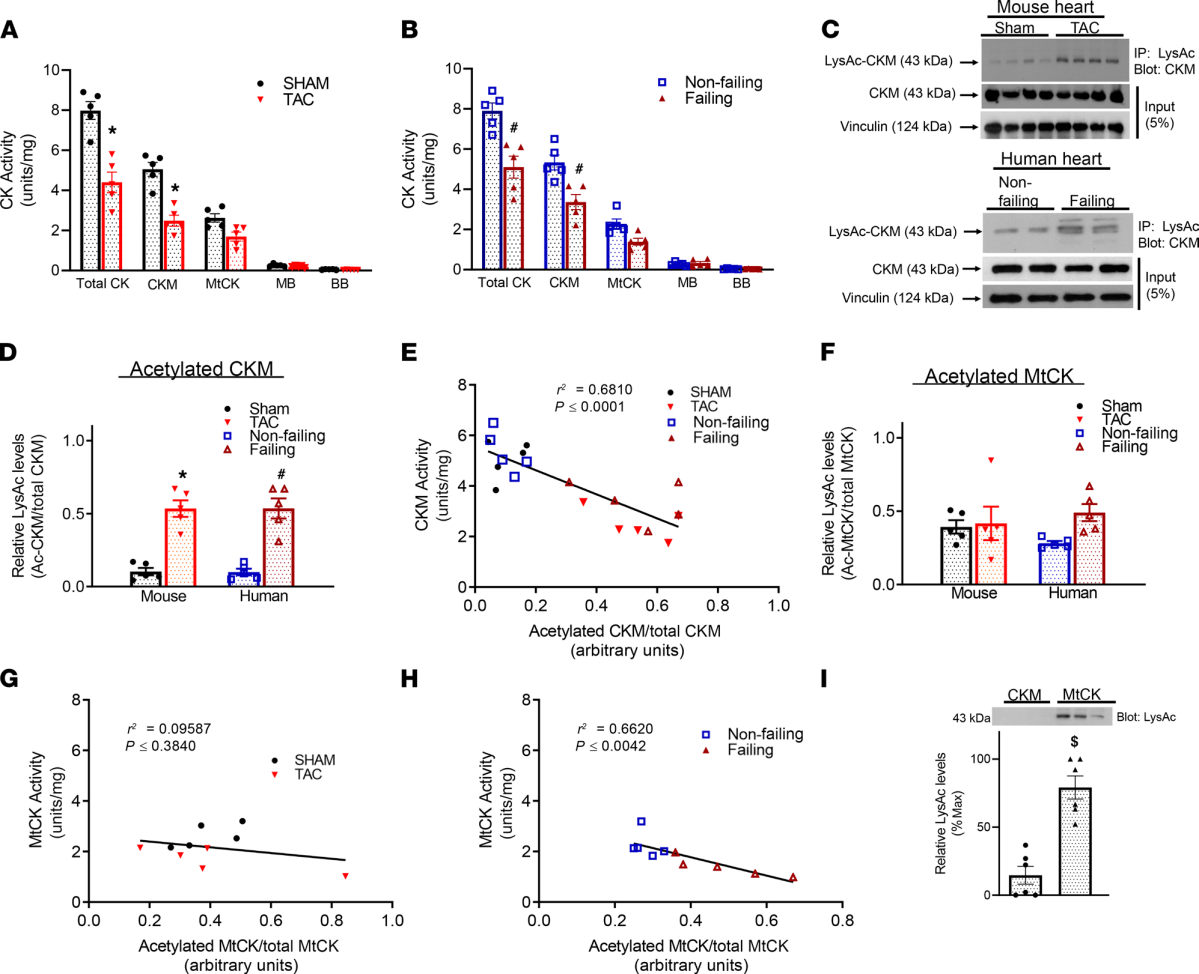
Lysine acetylation level on creatine kinase negatively correlates with its activity in heart failure. (**A** and **B**) Activity of CK isoforms were determined in myocardial samples from sham and TAC murine hearts (**A**) or from heart tissue from nonfailing and failing human hearts (**B**); *n =* 5 per group. (**C**) Representative immunoblots of total protein content and acetylation level of CKM in murine and human failing heart. (**D**) The ratio of acetylated CKM to total CKM protein content in murine and human failing hearts; *n =* 5 for all groups. (**E**) Correlation curve between the activity level of CKM isozyme and its acetylation level in murine and human heart failure samples. A linear regression analysis was used; *n =* 5 per group. (**F**) The ratio of acetylated MtCK normalized to total MtCK protein level in failing hearts; *n =* 5 per group. (**G** and **H**) Graphs showing the relation between the activity of MtCK and its acetylation level in mouse (**G**) and human (**H**) heart failure samples. A linear regression analysis was used; *n =* 5 per group. (**I**) Acetylation level of murine CKM and MtCK were compared using an anti–acetyl-lysine–specific antibody; *n =* 6 per group. All data presented as mean ± SEM. *P* value calculated by one-way ANOVA followed by Tukey post hoc analysis (**A**, **B**, **D**, and **F**) or by Student’s *t* test (**I**). *P < 0.05 versus sham, ^#^*P* < 0.05 versus nonfailing, ^$^*P* < 0.05 versus CKM.

We determined the relationship between CK activity and its protein level in human failing hearts due to ischemic or dilated cardiomyopathy (Supplemental Table 1; supplemental material available online with this article; https://doi.org/10.1172/jci.insight.144301DS1) and in a mouse model of heart failure induced by transverse aortic constriction (TAC) for 12 weeks (Supplemental Figure 1, A–C). As expected, heart failure in mice and men led to a significant reduction of the CK activity with predominant decreases in the activities of CKM and MtCK (Figure 1, A and B). Downregulation of CK activity was not associated with any changes in the protein level of CKM or MtCK in either human or mouse failing hearts (Figure 1C and Supplemental Figure 1, D–F). However, acetylation levels of CKM protein were significantly higher in the failing hearts (Figure 1, C and D), and protein acetylation was negatively correlated with CKM activity (Figure 1E and Supplemental Figure 1G). Compared with CKM, MtCK showed a higher basal level of acetylation in nonfailing hearts (Figure 1I). A moderate increase of MtCK acetylation was observed in human failing hearts but not in mouse TAC hearts (Figure 1F and Supplemental Figure 1E). Consistently, a negative correlation of MtCK acetylation and its activity was found in human hearts but not in mice (Figure 1, G and H, and Supplemental Figure 1, H–J). Taken together, these results suggest that protein acetylation is a potential mechanism regulating CK activity. Furthermore, acetylation of CK, especially CKM, could be a mechanism for the downregulation of CK reaction in the failing hearts.

### Acetylation reduced enzymatic activity of murine and human CKM.

**Figure 2:**
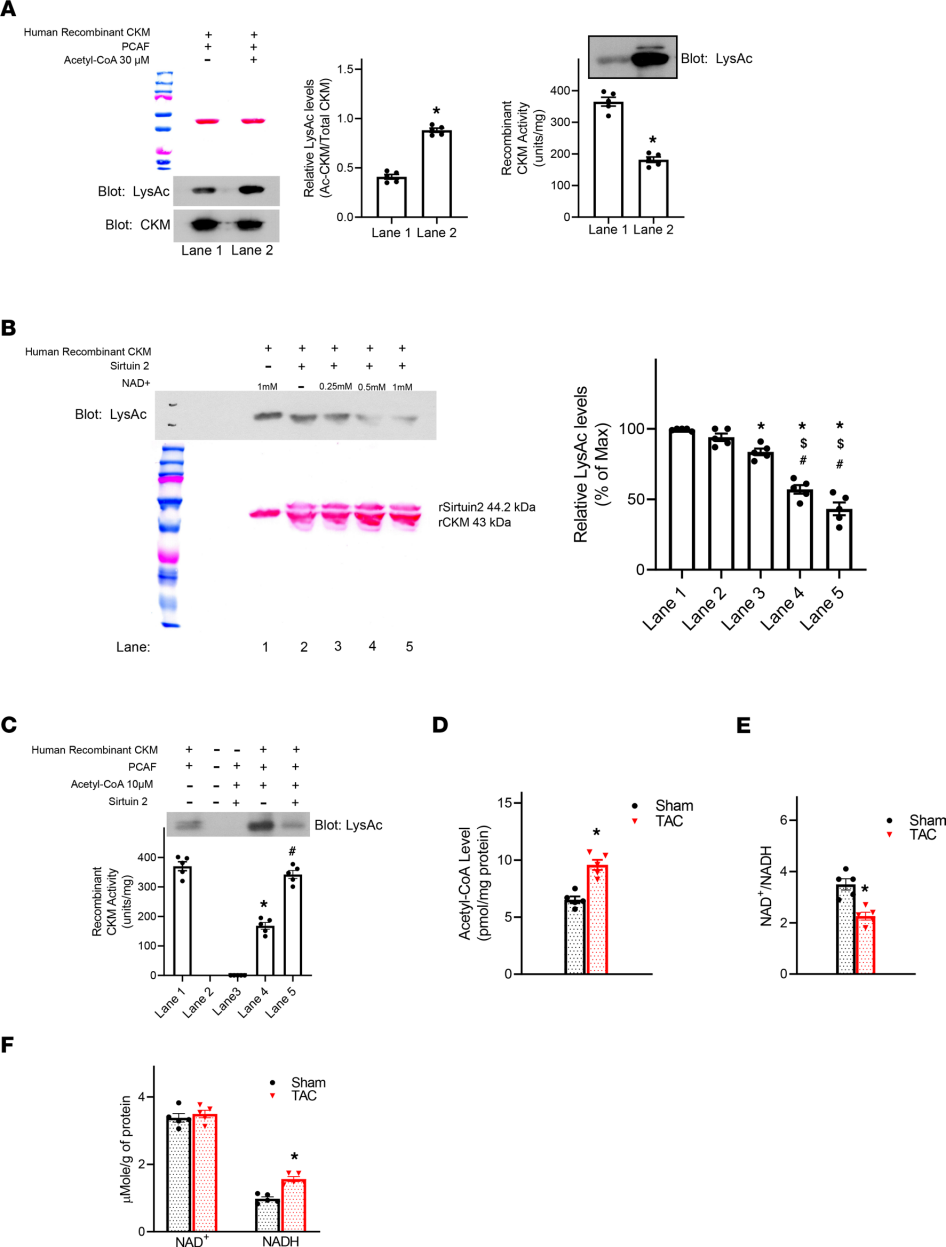
Acetylation reduced enzymatic activity of human CKM. (**A**) Representative immunoblot (left) and quantification (middle) showing increased acetylation of human recombinant CKM (rCKM) by PCAF; *n =* 5 per group. Ponceau S staining demonstrates equal protein loading. Activity of rCKM (right) from aliquots of lane 1 and lane 2 and representative immunoblot (top) of rCKM acetylation level; *n =* 5 per group. (**B**) Acetylation level of rCKM was detected in the absence (lane 1) or presence of Sirt2 (lanes 2–5). In lanes 2–5, NAD^+^ was added at increasing concentrations from 0–1 mM and deacetylation of rCKM assessed by WB; *n =* 5 per group. (**C**) Activity of rCKM was determined in aliquots from non–in vitro acetylated rCKM (lane 1), unloaded lane (lane 2), no rCKM (lane 3), PCAF-mediated acetylated rCKM (lane 4), and Sirt2 deacetylated aliquots (lane 5); *n =* 5 per group (**D** and **E**) Concentration of acetyl-CoA (**D**) and NAD^+^, NADH, and NAD^+^/NADH ratio (**E**) were determined in TAC-stressed hearts; *n =* 5 for all groups. Data represent *n =* 5 for all groups and are shown as mean ± SEM. *P* values were calculated by 1-way ANOVA followed by Tukey post hoc analysis (**B** and **C**) or by Student’s *t* test (**A**, **D**, **E** and **F**). *P < 0.05 versus lane 1 (**A**, **B**, and **C**) or versus sham (**D**, **E**, and **F**). ^#^*P* < 0.05 versus lane 2 (**B**) or lane 4 (**C**), or ^$^*P* < 0.05 versus lane 3 (**B**).

To investigate the causal relationship between CK acetylation and activity, we incubated human recombinant CKM (rCKM) with the acetyltransferase p300/CREB binding protein–associated factor (PCAF) and acetyl-CoA. The treatment resulted in a 1.9-fold increase in the relative acetylation level of rCKM and a ~50% decline in rCKM activity (Figure 2A). The acetylation level could be reverted by adding recombinant sirtuin 2 (Sirt2), which also restored CKM activity (Figure 2, B and C). Of note, the deacetylation by Sirt2 was dependent on the presence of NAD^+^ in a dose-dependent manner (Figure 2B). Taken together, the data substantiate the causality of protein acetylation in determining CK activity. Moreover, they suggest that such a regulatory mechanism was influenced by acetyl-CoA level and NAD^+^-dependent deacetylation.

We found TAC hearts to have marked increases in tissue acetyl-CoA concentration and significant reductions in the NAD^+^/NADH ratio (Figure 2, D–F). Previous work has shown spontaneous chemical reactivity to occur between surface-exposed lysine residues and acetyl-CoA on nonhistone proteins (18). There is some evidence that this occurs in heart failure where higher levels of tissue acetyl-CoA have been reported and associated with an overall increase in nonhistone protein acetylation (14, 15). This raises the possibility that surface-exposed lysine residues on CKM are particularly vulnerable to expansion of the acetyl-CoA pool or imbalanced NAD^+^/NADH ratio in failing heart.

**Figure 3:**
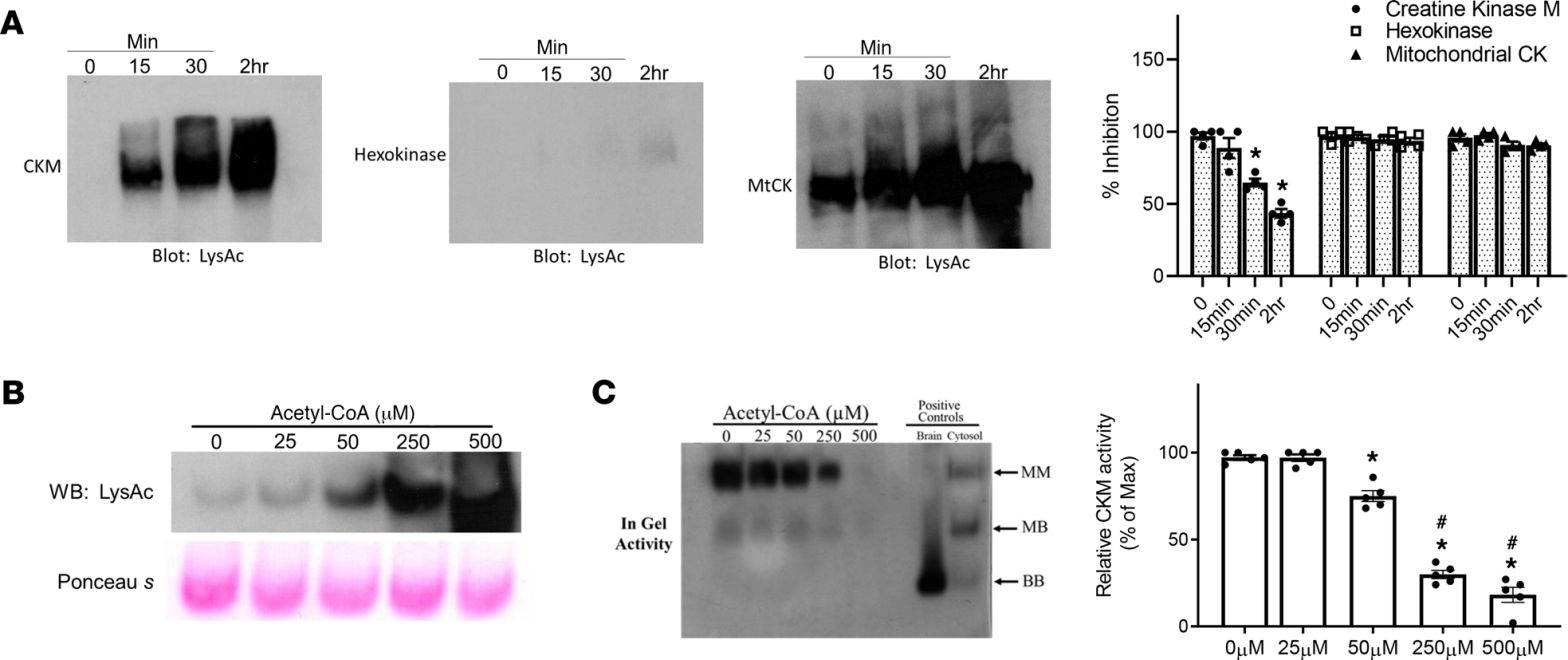
Acetylation reduced enzymatic activity of murine CKM but not MtCK. (**A**) CKM, MtCK, and hexokinase (HK) were incubated in 0.5 mM acetyl-CoA for either 15 minutes, 30 minutes, or 2 hours, and acetylation level or enzymatic activity were determined at each time point; *n =* 5 per group. (**B** and **C**) Incubation of murine CKM in acetyl-CoA (0–500 μM) and acetylation level assessed (**B**) or enzymatic activity (**C**) determined by in gel activity assay; *n =* 5 for all groups. Data represent *n =* 5 for all groups and are shown as mean ± SEM. *P* values were calculated by 1-way ANOVA followed by Tukey post hoc analysis (**A** and **C**). *P < 0.05 versus 0. ^#^*P* < 0.05 versus 25 μM (**C**).

Next, we tested the sensitivity and specificity of acetylation-mediated regulation of enzyme activity using CKM and MtCK enriched from mouse heart muscle. By incubating the proteins in 0.5 mM acetyl-CoA at increasing lengths of time, we were able to achieve higher levels of acetylation for both CKM and MtCK (Figure 3A). Graded increases of CKM acetylation resulted in dose-dependent inhibition of CKM activity, demonstrating a causal relationship between protein acetylation and loss of activity (Figure 3, A–C). Mass spectrometry (MS) analysis confirmed the increased acetylation of enriched murine CKM (Supplemental Table 3).

Unlike CKM, incubation with acetyl-CoA did not change the activity of MtCK or hexokinase (HK; Figure 3A). The inability of acetyl-CoA to change HK acetylation or activity suggests that protein modification by acetyl-CoA processed selectivity. On the other hand, MtCK presented a high degree of acetylation before incubation with acetyl-CoA, and its activity appeared to be insensitive to further acetylation (Figure 1, G–I, and Figure 3A). It has been shown that the alkaline pH environment and higher concentration of acetyl-CoA in mitochondria promotes nonenzymatic acetylation of mitochondrial protein under physiological conditions (18, 19). These findings are consistent with our observation that the relationship between MtCK acetylation and activity was more variable, suggesting that this mechanism is less robust for the regulation of MtCK activity, especially in mice (Figure 1, F–I).

### Lysine acetylation disrupts salt bridge formations between CKM monomers.

**Figure 4:**
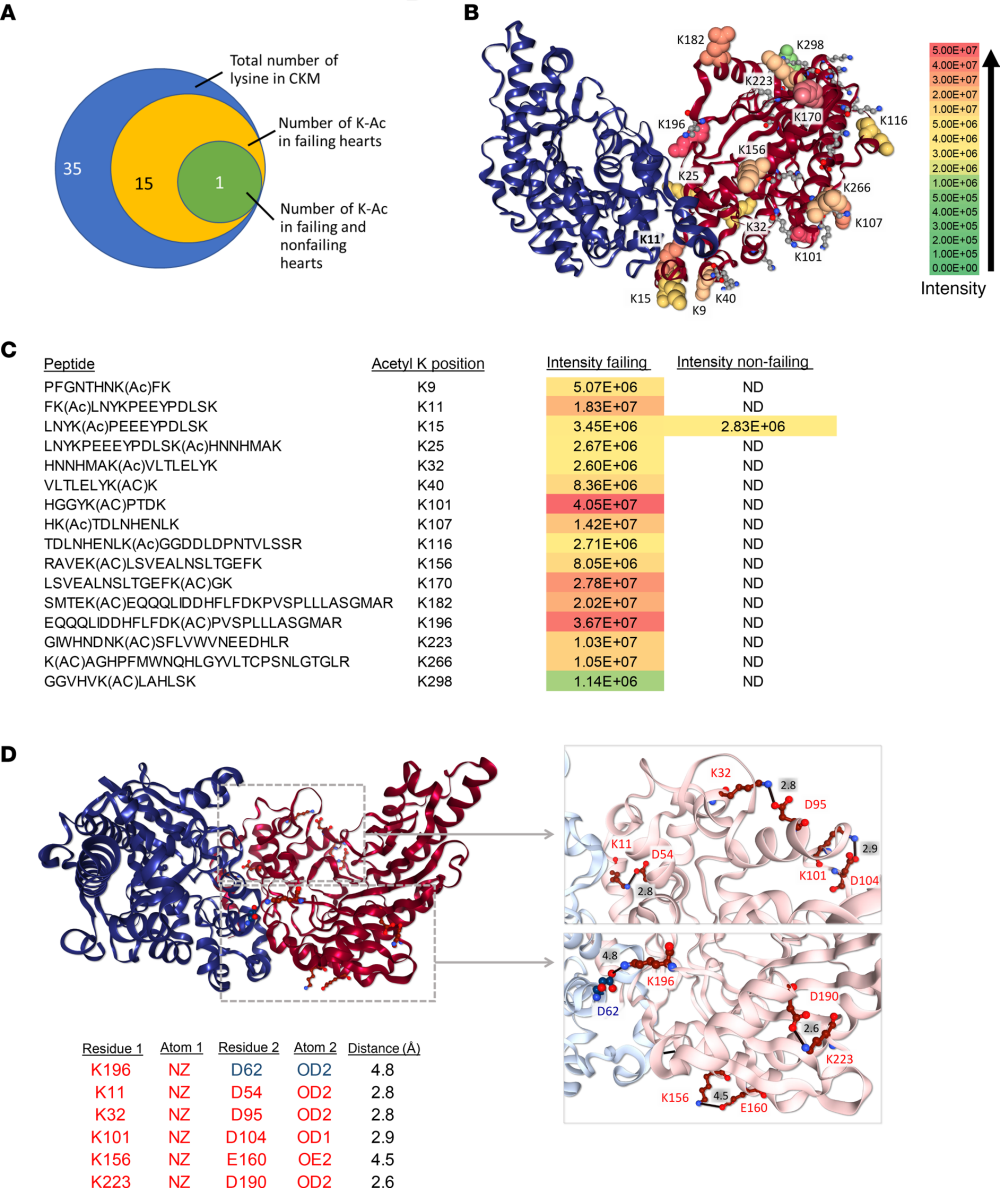
Lysine acetylation disrupts salt bridge formations between CKM monomers. (**A**) Venn diagram showing number of overlapping acetylated lysines in nonfailing and failing human heart CKM. (**B** and **C**) Identified acetylated lysine residues in failing heart are mapped to the CKM structure based on the rabbit structure PDB 1U6R (**B**); heatmap depicting relative abundance of acetylation at each lysine residue identified in nonfailing and failing heart presented as intensity and the corresponding peptides listed in **C**. (**D**) Total salt bridge formations involving acetylated lysine residues from failing heart. Red text indicates intrachain and blue text represents interchain salt bridges.

Human CKM has 35 lysine residues in each monomer, representing 9.2% of the 381–amino acid polypeptide (20). Using MS, we found ~46% of those lysine residues to be acetylated in failing hearts compared with just ~3% in nonfailing hearts (Figure 4A). Furthermore, acetylation level at each lysine residue was dramatically increased in heart failure (Figure 4, B and C). Previous work has reported increased CK acetylation in murine and human failing hearts, but its functional consequence has not been investigated (14, 16). To elucidate the mechanism by which acetylation impacts CK activity, we mapped acetylated lysine residues to a structural model of CKM based on the rabbit CKM structure PDB 1U6R (Figure 4B). It became apparent that conserved lysines (Supplemental Figure 2) located in regions of the protein that have been reported to support proper folding within and between monomer subunits and are critical for dimer formation (21) were also heavily acetylated in failing heart samples.

CKM is a very stable dimer that only disintegrates into monomers under harsh conditions or when mutated (21). It is known that acidic residues located in the N-terminal domain support communication between subunits by forming hydrogen bonds, van der Waals contacts, and salt bridges formed between positive residues such as lysine (K) and arginine (R) and negative residues such as aspartic acid (D) and glutamic acid (E) (20, 21). Contact areas between subunits of CKM are known to form polar interactions between negative residues — e.g., E18, D53, and D61 from 1 subunit and positive amino acids residues (R and K) on a second subunit (22). Mutational studies have shown that disruption of these residues in the dimer interface cause generation of monomeric CKM with reduced catalytic activity (22). The importance of these electrostatic interactions is highlighted by the human mutation in CKM (D54G; ref. 23). Substitution of aspartic acid with glycine removes the salt bridge between D54 and R148, which results in destabilization of the dimer interface and generation of monomeric CKM with reduced activity (23, 24). On the basis of these studies, we hypothesized that acetylation of lysines in this dimer interface region could neutralize the positive charge and disrupt multiple salt bridge formations, resulting in destabilization between monomeric subunits. Using XLinkDB structure view and a 5 Å cutoff for detecting salt bridges, we identified a total of 38 salt bridge formations in CKM (Supplemental Table 4). Of these, 5 acetylated lysines in failing heart (K11, K32, K101, K156, and K223) were found to form salt bridges within the monomer in a distance less than 5 Å with D54, D95, D104, E160, and D190, respectively (Figure 4D). Intrasalt bridges are critical for maintaining proper folding within the monomer, and disruption of monomer structural stability could ultimately lead to loss of dimer interaction. The intraprotein K11-D54 charge interaction could be also be important for dimer formation if it is either competitive with R148 or helps position D54 to interact with R148. In addition, we identified K196 as acetylated in failing heart and forming an interchain salt bridge with D62 (Figure 4D). The results suggest that positive charge of lysines could be neutralized by acetylation, thus disrupt salt bridge formation with negatively charged residues, resulting in reduced CKM dimer formation.

### Acetylation reduced CKM activity by preventing dimer formation.

**Figure 5:**
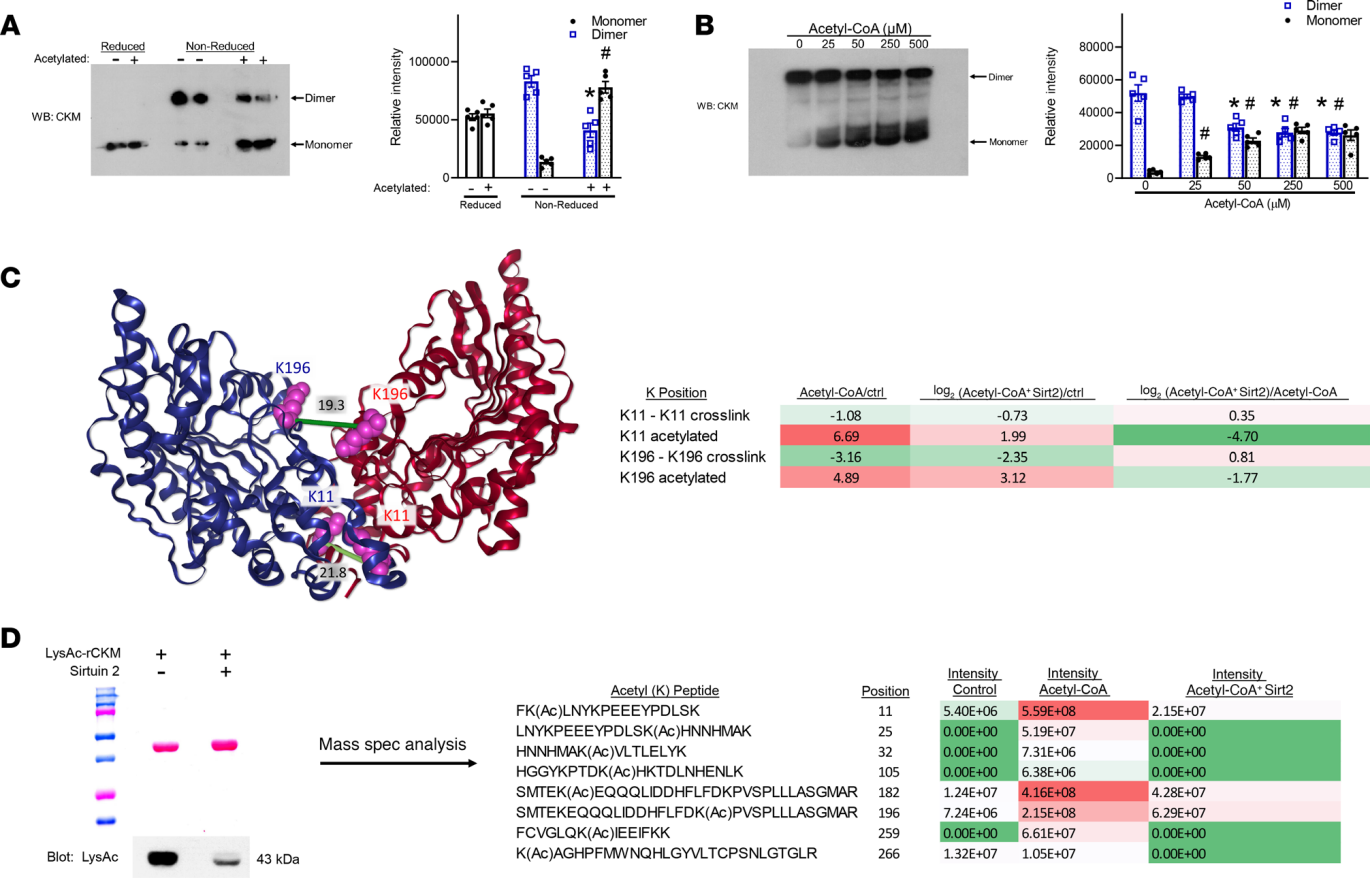
Acetylation of human recombinant CKM disrupts dimer formation. (**A**) Nonacetylated and acetylated human rCKM was prepared under reducing and nonreducing conditions and run on native-PAGE prior to Western transfer and immunoblotting with anti-CKM antibodies. Representative immunoblot (left) to determine dimeric versus monomeric CKM. Statistical analysis (right) of densitomeric measurements of monomer and dimer CKM; *n =* 5 per group. (**B**) rCKM was incubated over a range of acetyl-CoA concentration (0–500 μM) prior to being run on native-PAGE. Dimeric versus monomeric CKM was determined; *n =* 5 per group. (**C**) Crosslinking mass spectrometry (XL-MS) revealed distance constraints between crosslinked lysine residues and obtained information on the structure of the rCKM dimer mapped to PDB 1U6R. XL-MS analysis of rCKM found the homodimer links K196-K196 and K11-K11 were heavily disrupted with acetylation of rCKM. Sirt2 deaceytlation restored the K196-K196 and K11-K11 homodimer crosslink back toward control (right). (**D**) MS analysis of in vitro acetylated rCKM compared with Sirt2 deacetylated rCKM. Data shown as mean ± SEM; *n =* 5 per group. *P* values calculated by 1-way ANOVA followed by Tukey post hoc analysis. *P < 0.05 versus nonacetylated rCKM dimer, ^#^*P* < 0.05 versus nonacetylated Monomer rCKM (**A** and **B**).

To examine the possibility that acetylation disrupts the CKM homodimer, we subjected in vitro acetylated and nonacetylated human rCKM to native gel electrophoresis. When rCKM is in vitro acetylated, there is a 5.6-fold increase in the monomeric band in the native gel, with a 2.1-fold decrease in dimer band (Figure 5A). As the concentration of acetyl-CoA is increased, a dose-dependent increase in monomer formation was seen in native gels, and it corresponded with the downregulation of enzymatic activity (Figure 5, B and C). To obtain structural information of rCKM under these conditions, we used quantitative crosslinking MS (XL-MS) using a protein interaction reporter (PIR) crosslinker, biotin-aspartate proline-PIR n-hydroxyphthalimide (BDP-NHP; ref. 10). This allowed us to obtain distance constraints between crosslinked lysine residues and obtain information on the structure of the rCKM dimer (Figure 5C and Supplemental Figure 3). Among the 138 crosslinks obtained in rCKM (Supplemental Table 5), we found 2 crosslinks between lysines on Chain A and lysines on Chain B (K196A-K196B and K11A-K11B) (Figure 5C). Crosslinked residue pairs K196-K196 and K11-K11 are within the distance of 19.8 Å, and 21.8 Å, respectively, consistent with the homodimer configuration of CKM. In acetylated rCKM, we found crosslink K196-K196 to be reduced 9-fold and K11-K11 to be reduced 2-fold, indicating monomer generation (Figure 5C). Interestingly, after Sirt2 deacetylation of acetylated rCKM, the K196-K196 and K11-K11 crosslinks across the dimer interface were increased by 1.8-fold and 1.3-fold, respectively. This indicates restoration of the rCKM homodimer (Figure 5C). To determine if lysines identified in our salt bridge analysis (Figure 4D) are also deacetylated by Sirt2, we performed MS analysis of in vitro–acetylated rCKM and compared it with Sirt2-treated rCKM (Figure 5D). Lysine residues 11, 32, and 196 are robustly deacetylated by Sirt2, and we hypothesize that this allows the K11-D54, K196-D62, and K32-D95 salt bridges to reform in vitro.

**Figure 6:**
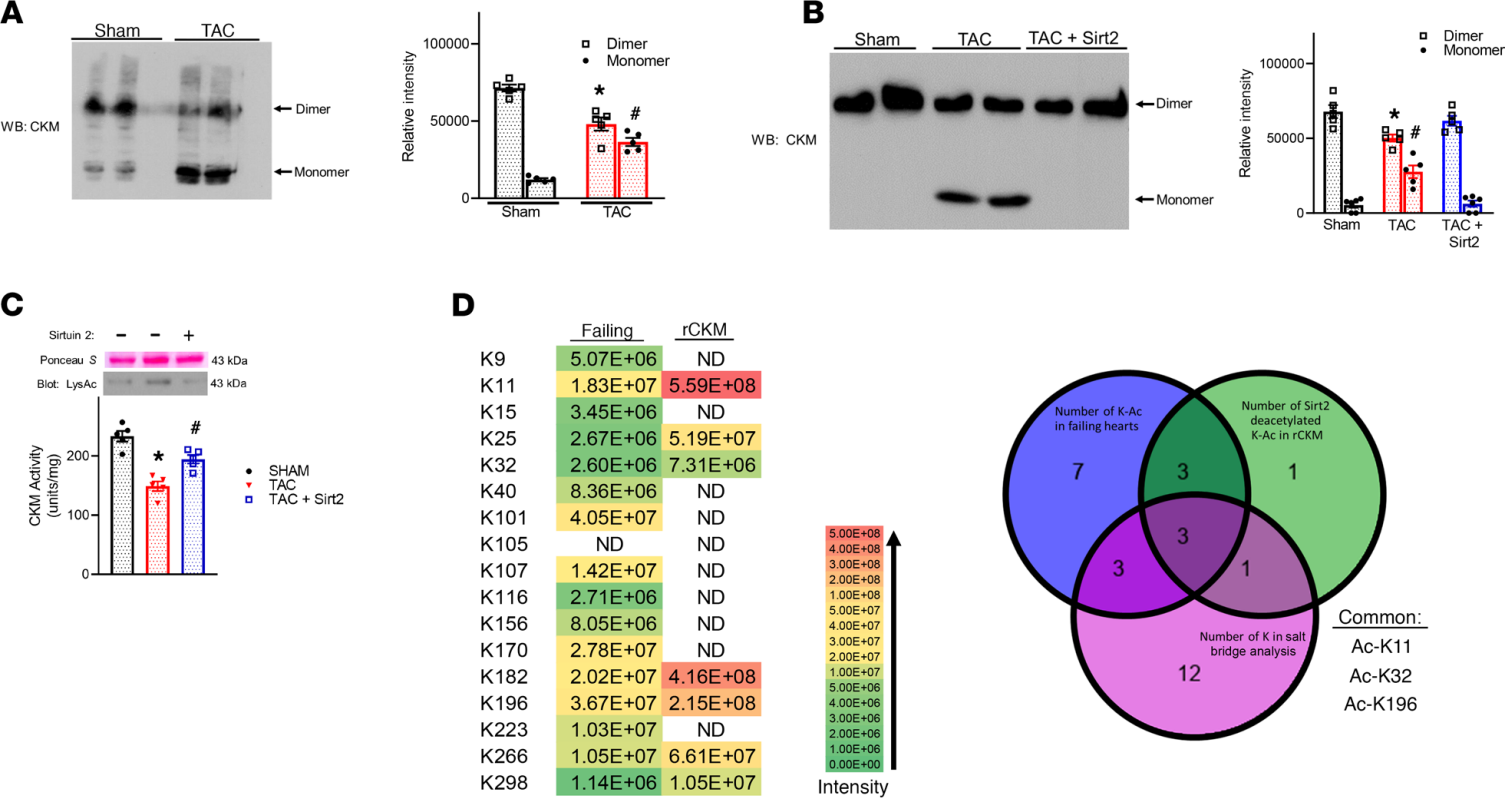
Acetylation reduced CKM activity by preventing dimer formation in the failing heart. (**A**) Cardiac lysates from sham and TAC-stressed hearts were run on native-PAGE. Representative immunoblot (left) of dimeric versus monomeric CKM. Statistical analysis (right); *n =* 5 each group. (**B** and **C**) Enriched CKM from TAC-stressed hearts was incubated with ± Sirt2 for an in vitro deacetylation reactions prior to native-PAGE to determine dimeric versus monomeric CKM (**B**) or aliquots were used to determine specific activity of CKM (**C**). (**D**) Acetylation sites of failing heart CKM compared with reversible in vitro acetylation sites on human recombinant CKM. Venn diagrams showing hyperacetylated lysine residues identified in failing hearts that also participate in salt bridge formations and were sensitive to deacetylation by Sirt2. Data shown as mean ± SEM; *n =* 5 per group. *P* value calculated by 1-way ANOVA followed by Tukey post hoc analysis. *P < 0.05 versus sham dimer, ^#^*P* < 0.05 versus sham monomer (**A** and **B**) or *P < 0.05 versus sham, ^#^*P* < 0.05 versus TAC (**C**).

We next asked if we could observe monomer generation in failing mouse hearts. We found that TAC increased the monomer of CKM by 2.8-fold and decreased dimer content by 1.4-fold in the mouse hearts (Figure 6A). Furthermore, in vitro deacetylation of CKM from TAC hearts by Sirt2 decreased monomer detected in native gels by 3.5-fold (Figure 6B) and could also restore enzyme activity in the failing mouse heart back to control levels (Figure 6C). Among the hyperacetylated lysine residues identified in the failing hearts, 6 participated in salt bridge formations and 6 were sensitive to deacetylation by Sirt2 (Figure 6D). The 3 lysines represented in all pools (K11, K32, K196) are likely key to the regulation of CKM activity by acetylation (Figure 6D). Taken together, these data show that hyperacetylation caused a shift from CKM dimers to monomers in failing hearts, resulting in lower enzymatic activity.

## Discussion

In the present study, we reveal a potentially novel mechanism in the regulation of CK activity in human heart failure. We propose that acetylation of CKM during the development of heart failure decreased its enzymatic activity and, hence, negatively impacts phosphotransfer via the CK reaction in failing hearts. For decades, clinical heart failure studies have shown reproducible reductions in myocardial CK activity and CK reaction flux, but the exact mechanisms for the downregulation have remained elusive and underresearched (1, 2, 8, 12). The results presented here show that CKM undergoes reversible acetylation in heart muscle, which is severely increased in failing hearts and negatively correlates with CK activity. Furthermore, we provide in vitro evidence that increasing the acetyl modification on CKM can cause a disruption of salt bridge formations within and between the M monomers, which ultimately result in destabilization of the MM homodimer. Because the same acetyl sites can be deacetylated by Sirt2 with reformation of the MM homodimer, we speculate that CKM could be enzymatically regulated by Sirt2 in vivo. In support of this occurring, we found considerable increases in monomeric CKM in failing heart samples that could be reformed into homodimers by Sirt2 treatment.

Although increased protein acetylation has been found in the failing hearts of animal models and human patients, the underlying mechanisms are less clear. The present results suggest that increased availability of acetyl-CoA and reduced deacetylation by sirtuin could both contribute to hyperacetylation of CKM. In support of this notion, proteomic comparisons reveal a significant overlap in hyperacetylated peptides from failing hearts and those affected by in vitro acetylation and deacetylation reactions using acetyl-CoA and Sirt2, respectively, in vitro. It is known that mitochondria generate and maintain acetyl-CoA concentrations (0.1–1.5 mM) many times higher than that of cytosol (15–50 μM; refs. 19, 25). The current study shows nonenzymatic acetylation of CKM by acetyl-CoA in a dose-dependent manner, and CKM could be acetylated in vitro at a concentration of 50 μM (high end of the physiological range) with decreased activity level. Furthermore, we find a higher level of acetyl-CoA in the TAC heart, consistent with a previous report in human failing hearts (26). Notably, these measurements of acetyl-CoA level do not distinguish subcellular pools; thus, they cannot provide unequivocal evidence of increased acetyl-CoA in the cytosol. Assessment of subcellular acetyl-CoA pools is technically challenging and is, therefore, a limitation we are unable to overcome.

Defective sirtuin function is likely another mechanism for increased acetylation of CKM in the failing heart. In support of this, Sirt2 protein expression is shown to be severely decreased in angiotensin-induced heart failure, and Sirt2-KO worsened the outcome in response to chronic pressure overload by TAC (27). Furthermore, decreases in NAD^+^ level and the NAD^+^/NADH ratio have been demonstrated in multiple heart failure models and human failing hearts, and the decreases are shown to impair sirtuin deacetylase activity (14, 15, 28). In this light, our MS analysis has identified a subset of specific lysine residues that are hyperacetylated in failing heart and also undergo deacetylation by Sirt2 in vitro. This dataset will allow for a more targeted approach in future studies to test if manipulation of CK acetylation in vivo influences heart function and contributes to the development or transition to heart failure.

Unlike in CKM, acetylation appears to play a minor role in the downregulation of mtCK activity in heart failure. MtCK is highly acetylated in the normal heart, likely due to the exposure to high acetyl-CoA level in mitochondria. Changes of mtCK acetylation are not robust in the failing heart, and mtCK enriched from murine heart is relatively resistant to further acetylation in vitro. MtCK is known to form higher oligmeric structures (Octamers); thus, it may not be susceptible to acetylation modification, as CKM is. Along the similar line, a recent study by Davidson et al. (29) shows that hyperacetylation of mitochondrial protein does not impair cardiac bioenergetics, suggesting that acetylation has a limited effect on the function of mitochondrial proteins. It is, thus, possible that distinct mechanisms are responsible for the decreased activity of CKM and mtCK in heart failure. Since mtCK is extremely sensitive to damage caused by reactive oxygen species (30), mitochondrial dysfunction and increased oxidative stress in heart failure could play a primary role in damaging mtCK.

This study has several limitations. Due to a limited supply of human heart tissue, the monomer and dimer composition of CKM was only analyzed in mouse failing hearts. Nevertheless, we found that in vitro acetylation of recombinant human CKM reduced dimer formation. MS analysis showed a significant overlap of lysine residues, contributing to dimer formation in rCKM and acetylated lysines in human failing hearts. These findings strongly suggest that acetylation of CKM can inhibit dimer formation in human failing hearts. Despite strong in vitro evidence, the study did not provide in vivo data directly linking CK acetylation with myocardial energetics and heart failure. Previous studies by us and others have shown that manipulation of overall protein acetylation is associated with changes of cardiac energetics and function (15, 27). However, a large number of proteins change acetylation in these studies, rendering it difficult to single out the role of CK acetylation. The present study has identified a small number of lysine residues critical for regulating CK activity through acetylation. Future studies targeting these lysines have the potential to address the causal role of CK acetylation in vivo. Additional studies are also needed to determine whether CKM acetylation mechanism can be utilized to develop heart failure therapy.

In summary, we found an inverse relationship between the activity and acetylation of CKM in human and mouse failing hearts. Hyperacetylation disrupted MM homodimer formation and reduced enzymatic activity, which could be reversed by Sirt2 treatment. These results reveal a potentially novel mechanism for the downregulation of CK activity in the failing heart. They also provide an opportunity to target cardiac bioenergetics in heart failure.

## Methods

### Animal studies, surgical procedures, and echocardiography

WT mice (129S1/SvImJ, stock no. 002448) were purchased from the Jackson Laboratory. Male mice (3–4 months old) underwent TAC or sham surgery. Briefly, mice were anesthetized with an i.p. injection of 130 mg/kg ketamine and 8.8 mg/kg xylazine in saline. Mice were intubated with 20 G cannula and ventilated 140 breaths per minute by a small animal TOPO ventilator (Kent Scientific). The aortic arch was exposed via a left thoracotomy and by carefully separating the thymus. A constriction of the transverse aorta was generated by tying a 6-0 Ethilon ligature against a 27-gauge blunt needle around the aorta between the brachiocephalic and left common carotid arteries. Promptly, the needle was removed and the chest and skin were closed by a 5-0 polypropylene suture. The animal was removed from ventilation and kept on a heating blanket during recovery from anesthesia. SR buprenorphine (0.05 mg/kg) was administered s.c. for analgesia. Sham-operated mice underwent all the same procedures as TAC mice, excluding the constriction of the aorta. All mice were monitored every 12 hours during the first 72 hours after surgery, followed by daily visits over the next 4 weeks.

Echocardiography of hearts was performed 12 weeks after surgery using the VEVO 2100 high-frequency, high-resolution digital imaging system (VisualSonics) equipped with a MS400 Microscan Transducer on anesthetized mice (1%–2% isofluorane in 95% oxygen). Measurements were made when the heart rate was within 500–550 bpm. Cardiac function and geometry measurements were measured in parasternal short axis view. M-mode images were used for analysis and calculated by the average of at least 3 cardiac cycles, and they were carried out in a blind fashion. Hearts were harvested at 12 weeks after surgery for subsequent experiments and biochemical assays.

### Antibodies and recombinant proteins

#### Antibodies.

Antibodies used in this study included the following: acetyl-lysine antibody (Cell Signaling Technology, 9441), anti-CKMT2 antibody (MilliporeSigma, SAB2100437), anti-CKM (Santa Cruz Biotechnology Inc., G-9, sc-365046), and anti-vinculin (Cell Signaling Technology, 13901S).

#### Recombinant proteins.

Human CK MM (rCKM) was purchased from R & D Systems (catalog 9070-CK) and from Abcam (catalog ab73652). Recombinant Sirt2 was purchased from Cayman Chemical (catalog 10011191), PCAF (Cayman Chemical, 10009115), HK from Saccharomyces cerevisiae (MilliporeSigma, H4502), and glucose-6-phosphate dehydrogenase from Leuconostoc mesenteroides (Sigma G5885).

### Western blot

For Western blot procedures, cardiac tissues were homogenized with RIPA buffer (Thermo Fisher Scientific, RIPA buffer 89900) and protease inhibitors (Roche, 11-836-170-001) using a bullet blender (Next Advance, BBX24B) at 4°C for 10 minutes. Protein concentrations of supernatants were collected after centrifugation (20,000*g*, 4°C, 20 minutes) and quantified using Pierce BCA Protein Assay Kits and Reagents (catalog 23225). Protein lysates in Lammeli sample buffer were loaded to 10 % SDS-PAGE, transferred to PVDF membrane, and then incubated at room temperature in Ponceau S (MilliporeSigma, P7170) for 10 minutes. Membranes were washed three times in distilled H_2_O and then scanned to determine protein loading. Membranes were then blocked with 5% BSA in TSBT for 1 hour at room temperature. Specific proteins were detected by specific antibodies listed above and corresponding secondary antibodies. Signals were visualized by HRP-derived chemiluminescence (Amersham ECL Western Blotting Detection System, RPN2106) and film. Protein levels were quantified by ImageJ (NIH).

### IP

In acetylation analysis with Acetyl-lysine–specific IP, 25 mg of human or murine cardiac tissue was homogenized in IP buffer (50 mM Tris-Hcl [pH 7.5], 10 mM Sodium phosphate, 50 mM Sodium Chloride, 0.1% Triton X-100, 1 mM EDTA, 10 μM Trichostatin A, 10 mM nicotinamide, protease inhibitors). Homogenized tissue was cleared by 600*g* centrifugation for 10 minutes (4°C), and supernatant was incubated for 1 hour on ice to release mitochondrial protein. The lysate was then incubated with Acetyl lysine antibody–conjugated to agarose (ImmuneChem, Burnaby ICP0388) or control immunoglobulin conjugated to agarose (Santa Cruz Biotechnology Inc., sc-2343) at 4°C overnight with mild agitation. Agarose bound with acetylated proteins or immune controls were washed gently with IP buffer, followed by centrifugation to pellet beads (1000*g*, 4°C, 5 minutes). This wash step was repeated 2 times, followed by 2 additional washes with cold PBS and then by centrifugation (1000*g*, 4°C, 5 minutes). Bound acetylated proteins were released with 0.1M glycine (pH 2.5; MilliporeSigma, 8898) for 10 minutes at room temperature and then neutralized with 2M Tris (pH 8.0; Bioston BioProducts, BM-325; MilliporeSigma, T1503). SDS loading buffer was added and samples were boiled for 5 minutes at 95°C. Samples were loaded to 10% SDS-PAGE gels for analysis.

### Enrichment of CKM from myocardial tissue

CKM was extracted from pooled heart tissue (~200 mg) in a 2-step protocol involving Blue Sepharose CL6B affinity chromatography (Amersham Biosciences, HiTrap Blue HP GE17-0413-01) and ion-exchange chromatography using Deae Sephadex-A50 Ion Exchange Chromatography Media (GE Healthcare, 17-0180-01) as previously described (31). Briefly, soluble protein from cytosolic fractions was obtained by homogenizing heart tissue with a dounce homogenizer, followed by centrifugation at 600*g* at 4°C for 10 minutes to pellet nuclear and 10,000*g* at 4°C for 10 minutes to pellet mitochondrial fractions. After initial homogenization, suspension was spun at 600*g* for 10 minutes and the pellet was discarded. The supernatant was then spun 12,000*g* for 10 minutes to pellet mitochondria. The mitochondrial pellet was discarded, and supernatant was spun again at 12,000*g* for 10 minutes. The supernatant (cytosolic fraction) was applied to a 5 mL Blue Sepharose affinity column. The column was washed with 30 mL of mobile phase (50 mM sodium phosphate [pH 5.8]), and protein was eluted with 30 mL of 50 mM sodium phosphate (pH 8.5). Fractions containing CKM, as determined by activity and immunoblotting, were pooled, diluted in 50 mM sodium phosphate (pH 5.8), and reapplied to the Blue Sepharose column. The column was washed in 30 mL of mobile phase (50 mM sodium phosphate [pH 5.8]), and CKM was eluted with 30 mL of 50 mM sodium phosphate (pH 8.5). The elute was then applied to cation-exchange chromatography Deae Sephadex A-50 column on a linear salt gradient from 50 to 480mM of NaCl. Peak CKM fractions were pooled, diluted in buffer, and then concentrated (Amicon) and saved for kinetic, immunoblot, and further experimental analyses.

### Enrichment of MtCK from myocardial tissue

Extraction of MtCK from heart muscle was performed in a 2-step protocol involving Blue Sepharose CL6B affinity chromatography (Amersham Biosciences, HiTrap Blue HP GE17-0413-01) and ion-exchange chromatography (Deae Sephadex A-50 Ion Exchange Chromatography Media, GE Healthcare, 17-0180-01) adapted from ref. 32. Briefly, the murine heart tissue was homogenized in buffer (220 mM mannitol, 70 mM sucrose, 10 mM HEPES [MilliporeSigma, H4034], 0.2 mM EDTA, 1 mM 2-mercaptoethanol [BME; MilliporeSigma, M7522], 10 μM Trichostatin A [catalog T8552], 10 mM nicotinamide [MilliporeSigma, N3376], protease inhibitors [pH 7.4]). The suspension was homogenized, and then centrifugation of the homogenate was performed for 10 minutes at 600*g* (all centrifugation steps were at 4°C for 20 minutes). The supernatant was centrifuged for 20 minutes at 12,000*g* to pellet mitochondria. After washing the crude mitochondrial pellet with the above buffer, the mitochondria were again pelleted with a centrifugation for 20 minutes at 12,000*g*. The enriched mitochondria fractions were pooled and then incubated overnight at 4°C in 50 mM sodium phosphate, 1 mM MgCl_2_, 1 mM BME, 0.2 mM EGTA (MilliporeSigma, E4378), 10 μM Trichostatin A, 10 mM nicotinamide, and protease inhibitors [pH 8.0], under slow stirring. The lysate was centrifuged at 10,000*g*, and it was resuspended and diluted in 50 mM sodium phosphate, 1 mM MgCl2, 1 mM BME, and 0.2 mM EGTA [pH 5.8] and run on the on Blue-Sepharose column. Then the column was rinsed with 30 mL of Blue-Sepharose buffer (pH 5.8) and subsequently with 30 mL of the same buffer changed to pH 8.0. Then MtCK was eluted with the above buffer supplemented with 10 mM ADP, and the elute was run over a ion-exchange chromatography column on a linear salt gradient from 50 to 480 mM of NaCl. Enriched MtCK fractions (between 190 and 240 mM NaCl) were pooled, concentrated, and used for downstream analysis.

### CK activity measurements and isozyme distributions

Murine or human heart muscle (10 mg) was homogenized at 4°C in 50 mM potassium phosphate buffer containing 1 mM EDTA (MilliporeSigma, ED2SC) and 1 mM β-mercaptoethanol (pH 7.4; final concentration, 5 mg tissue/mL). Aliquots were removed for protein assays. Triton X-100 (Perkinelmer Life and Analytical Science, N9300260) was then added to the homogenate at a final concentration of 0.1% and incubated at 1 hour on ice. Total CK activity was measured in homogenates spectrophotometrically via a coupled-enzyme assay (33, 34). Using the same samples, individual CK isoenzymes were separated by electrophoretic mobility on an agarose gel, and a coupled-enzyme assay was performed as described (33). Absolute activities for each isoenzyme were calculated by multiplying relative isoenzyme activity (measured by densitometry) with total CK activity. Enzyme activities were measured (units/mg protein).

rCKM, murine CKM, and murine MtCK activities were determined spectrophotometrically via a coupled-enzyme assay as described previously (34). HK (MilliporeSigma) activity was determined by enzyme coupled assay using glucose-6-phosphate dehydrogenase.

### Acetyl-CoA concentration and NAD^+^/NADH determination

Acetyl-CoA (MilliporeSigma, A2056) concentrations was determined in 10 mg of cardiac tissue using the PicoProbe Acetyl CoA Assay Kit (Fluorometric) per manufacture instructions (Abcam, ab87546). NAD^+^ and NADH were determined in 8 mg tissue using BioAssay Systems EnzyChrom NAD^+^/NAD Assay Kit (E2ND-100) per manufacture instructions.

### In vitro acetylation and deacetylation assays

For in vitro nonenzymatic acetylation reactions of murine CKM, MtCK, HK (MilliporeSigma), and human rCKM, enzymes were incubated in acetylation buffer (50 mM HEPES, 150 mM NACl [pH 7.4]) supplemented with the indicated concentrations of Acetyl-CoA (0 μM, 25 μM, 250 μM, or 500 μM) at time points of 15 minutes, 30 minutes, 2 hours, or 6 hours at 37°C with gentle rocking (400 rpm). For enzymatic in vitro acetylation reactions, the enzymes were incubated in the acetylation buffer supplemented with PCAF (Cayman Chemical, 10009115) plus 30μM Acetyl-CoA rocking (400 rpm) at 37°C for 1 hour.

For in vitro deacetylation reactions, enzymes were incubated in deacetylation buffer (50 mM Tris-HCl [pH 7.5], 150 mM NaCl, 1 mM MgCl2, 0.5 μM TSA) supplemented with Sirt2 (Cayman Chemical, 10011191) and NAD^+^ concentrations of 0 mM, 0.25 mM, 0.5 mM, or 1 mM rocking (400 rpm) at 37°C for 1 hour.

For downstream analysis after in vitro acetylation or deacetylaton reactions, the reactions were either: (a) diluted 10-fold in appropriate enzyme activity assay buffer for specific activity measurements, (b) diluted in SDS Lammeli sample buffer and boiled 95°C for 5 minutes for immunoblotting, (c) diluted in native-PAGE sample buffer and run on native gels, or (d) crosslinked per BDP crosslink protocol described below or liquid chromatography–MS (LC-MS) analysis as described below.

### LC-MS analysis of acetylated peptides and crosslinked peptides

Peptide samples were analyzed by LC–MS using an Easy-nLC (Thermo Fisher Scientific) coupled to a Q Exactive Plus mass spectrometer (Thermo Fisher Scientific). Peptides were loaded onto a 3 cm × 100 μm inner diameter–fused silica trap column packed with a stationary phase consisting of 5 μm Reprosil C8 particles with 120 Å pores (Dr. Maisch GmbH) with a flow rate of 2 μL/min of mobile phase consisting of solvent A (H_2_O containing 0.1% formic acid) for 10 minutes. Peptides were then fractionated over a 60 cm × 75 μm inner diameter fused silica analytical column packed with 5 μm Reprosil C8 particles with 120 Å pores by applying a linear gradient from 95% solvent A, 5% solvent B to 60% solvent A, and 40% solvent B over 120 minutes at a flow rate of 300 nL/min. Eluting peptides were ionized by electrospray ionization by applying a positive 2.2 kV potential to a laser pulled spray tip at the end of the analytical column. The mass spectrometer was operated using a top 20 data-dependent acquisition method with a resolving power setting of 70,000 for MS1 and 17,500 for MS2 scans. Additional settings include an AGC target value of 1 × 10^6^ with a maximum ion time of 100 ms for the MS1 scans and an AGC value of 5 × 10^4^ with a maximum ion time of 100 ms for the MS2 scans. Charge state exclusion parameters were set to only allow ions with charge states from 2+ to 8+ to be selected for MS2. Ions selected for MS2 were isolated with a 3 *m/z* window and fragmented by HCD using a normalized collision energy setting of 27. Ions for which MS2 was performed were then dynamically excluded from further selection for MS2 for 30 seconds.

Identification and quantification of acetylated peptide and protein Raw MS data were processed using MaxQuant software (ver.1.6.17.0). Acetylated peptides were identified using the integrated Andromeda search algorithm (35). Mouse samples were searched against Uniprot mouse protein sequence database (downloaded on April 28, 2018). Human samples were searched against Uniprot human protein sequence database (downloaded on April 28, 2018). rCKM data were searched against Uniprot Ecoli FASTA database (downloaded on August 6, 2019) spiked with rCKM sequence. The following parameters were used for searches: trypsin specificity with a maximum of 4 missed cleavages; carbamidomethylation of cysteine as fixed modification; oxidation of methionine, acetylation of protein N-term, and acetylation of lysine as variable modifications; instrument and MS/MS analyzer parameters used the default settings for Orbitrap; protein quantitation used only unmodified peptides and oxidized methionine; and all peptide and protein identifications filtered using 1% FDR cut-off from reversed sequence decoy database. Acetylated peptides were further filtered with localization probability score greater than 90%.

Crosslinked peptide samples were analyzed with the same LC-MS system described above using the same LC gradient conditions and mass spectrometer parameters with the following differences. The mass spectrometer was operated with a resolving power setting of 70,000 for MS1 and MS2 scans. Charge state exclusion parameters were set to only allow ions with charge states from 4+ to 8+ to be selected for MS2. Ions selected for MS2 were isolated with a 3 *m/z* window and fragmented by HCD using a normalized collision energy setting of 30.

### Salt bridge analysis

Salt bridge analysis was done using XLinkDB structure view and a 5 Å cutoff for detecting salt bridges.

### Clustal multiple sequence alignment and Venn diagrams

Multiple sequence alignment of creatine kinase M-type (KCRM) across species was run using Clustal Omega tool (https://www.ebi.ac.uk/Tools/msa/clustalo/) (36), and Venn diagrams were created using GeneVenn (http://genevenn.sourceforge.net/).

### Native gels

Murine cardiac lysates or human rCKM were run on 10% native-PAGE gels (1M Tris [pH 8.8], 30%Bis-Acrylamide, dH_2_O, 10% APS, TEMED; Thermo Fisher Scientific). For cardiac lysates, murine heart muscle (10 mg) was homogenized at 4°C in 50 mM potassium phosphate buffer containing 1 mM EDTA and 1 mM β-mercaptoethanol (pH 7.4; final concentration, 5 mg tissue/mL). Aliquots were removed for protein assays. Triton X-100 was then added to the homogenate at a final concentration of 0.1% and incubated at 1 hour on ice. Tissue lysates or CKM were further diluted in native-PAGE sample loading buffer (10% v/v glycerol, 0.0185% w/v Coomassie G-250) and then loaded into native-PAGE gel without prior heating.

Native-PAGE running buffer (50 mM Tris-Base, 50 mM MOPS, 0.0375% SDS [pH 7.8]) was prechilled to 4°C and kept at the temperature for the duration of the experiment. Samples were then loaded into the gel, and electrophoresis was conducted at constant voltage of 75 V for 20 minutes followed by 100 V for 1 hour at 4° C. Native gels were then transferred to PVDF membrane (100 V for 1 hour at 4°C) and blocked with 5% BSA (Boston BioProducts, P-753) in TSBT. Dimeric versus monomeric CKM were detected by specific antibodies listed above and corresponding secondary antibodies. Reduced samples of CKM in SDS Lammeli sample buffer were also included as positive controls on native-PAGE gels to verify monomer band.

### Rna extraction and quantitative PCR (qPCR)

Total RNA was isolated from frozen heart tissue using the RNeasy fibrous Kit (Qiagen). Omniscript reverse synthase and random hexamers were used for cDNA synthesis according to manufacturer guidelines. Real-time PCR was performed using SYBR green (Bio-Rad). Results of mRNA levels were reported as fold-change over control. Primers used include the following: ANP, forward 5′-ATTGACAGGATTGGAGCCCAGAGT-3′ and reverse 5′-TGACACACCACAAGGGCTTAGGAT-3′; and BNP, forward 5′-GCCAGTCTCCAGAGCAATTCA-3′ and reverse 5′-GGGCCATTTCCTCCGACTT-3′.

### Statistics

All data presented as mean ± SEM. Statistical analysis was performed with GraphPad Prism 8.3 (GraphPad Software). Normal distribution of the data was analyzed using a Shapiro-Wilk test. Comparisons between 3 or more groups were conducted by 1-way ANOVA followed by a Tukey post hoc analysis. For repeated measurements of multiple groups, 2-way repeated-measures ANOVA was performed. Comparisons between 2 groups was done by unpaired, 2-tailed Sudent’s *t* test. All results were tested at the *P* < 0.05 level of significance. For correlation graphs, linear regression analysis was run on GraphPad Prism 8.3.

### Study approval

#### Animal care.

All procedures involving animal use were performed with the approval of IACUC of the University of Washington.

#### Myocardial samples from nonfailing and failing human hearts.

Human left ventricular tissue were obtained from deidentified frozen myocardial samples. The failing heart tissue was obtained from discarded tissue at the time of left ventricular assist device implantation as described previously (37). Nonfailing heart tissue from individuals with no diagnosed cardiac disease was obtained previously from donor hearts deemed unsuitable for transplantation as described (37).

#### Animal care, surgical procedures, and echocardiography.

All procedures involving animal use were approved by the IACUC of the University of Washington.

## Author contributions

MAW, JC, JEB, and RT designed the experiments. MAW, JC, and OV performed the experiments. MAW, JC, XT, AK, JEB, and RT analyzed the data and wrote the manuscript.

## Supplementary Material

Supplemental data

Supplemental Table 5

## Version 1 02/08/2021

Electronic publication
