# Supplementary material for: Acetylation of muscle creatine kinase negatively impacts high-energy phosphotransfer in heart failure
Source: JCI Insight. 2021 Feb 8;6(3):e144301. doi: 10.1172/jci.insight.144301 (PMC7934860; doi:10.1172/jci.insight.144301)
Supplement: Supplemental data [file jciinsight-6-144301-s132.pdf]

Supplementary Methods

Animal care, surgical procedures, and echocardiography

All procedures involving animal use were approved by the Institutional Animal Care and Use Committee (IACUC) of the University of Washington. Wild-type mice (129S1/SvImJ Stock No: 002448) were purchased from Jackson Laboratories. Male mice (3-4 months old) underwent transverse aortic constriction (TAC) or sham surgery. Briefly, mice were anesthetized with an intraperitoneal injection of 130 mg/kg ketamine and 8.8 mg/kg xylazine in saline. Mice were intubated with 20 G cannula and ventilated 140 breaths per minute by small animal TOPO ventilator (Kent Scientific). The aortic arch was exposed via a left thoracotomy and by carefully separating the thymus. A constriction of the transverse aorta was generated by tying a 6-0 Ethilon ligature against a 27-gauge blunt needle around the aorta between the brachiocephalic and left common carotid arteries. Promptly the needle was removed and the chest and skin were closed by 5-0 polypropylene suture. The animal was removed from ventilation and kept on a heating blanket during recovery from anesthesia. SR buprenorphine (0.05 mg/kg) was administered subcutaneously for analgesia. Sham operated mice underwent all the same procedures as TAC mice excluding the constriction of the aorta. All mice were monitored every 12 h during the first 72 h post-surgery, followed by daily visits over the next 4 weeks.

Echocardiography of hearts was performed at indicated time after surgery using the VEVO 2100 high-frequency, high-resolution digital imaging system (VisualSonics) equipped with a MS400 Microscan Transducer on anesthetized mice (1%-2% isofluorane in 95% oxygen). Measurements were made when heart rate was within

500-550 bpm. Cardiac function and geometry measurements were measured in parasternal short axis view. M-mode images were used for analysis and calculated by the average of at least three cardiac cycles and carried out in a blind fashion. Hearts were harvested at 12-week post-surgery for subsequent experiments and biochemical assays.

Myocardial samples from non-failing and failing human hearts

Human left ventricular tissue were obtained from de-identified frozen myocardial samples. The failing heart tissue was obtained from discarded tissue at the time of left ventricular assist device implantation as described previously (1). Non-failing heart tissue from individuals with no diagnosed cardiac disease was obtained previously from donor hearts deemed unsuitable for transplantation as described (1).

Antibodies and recombinant proteins

Antibodies: Acetyl-lysine antibody (Cell Signaling #9441), Anti-CKMT2 antibody (Sigma-Aldrich SAB2100437), CKM (Santa Cruz, G-9 sc-365046), Vinculin (Cell Signaling #13901S).

Recombinant proteins: Human creatine kinase MM (rCKM) was purchased from R & D Systems (catalogue number: 9070-CK) and from Abcam (ab73652). Recombinant sirtuin 2 was purchased from Cayman chemical (Item number: 10011191), PCAF (Cayman Chemical Item No. 10009115), Hexokinase from Saccharomyces cerevisiae (Sigma H4502), and Glucose-6-phosphate Dehydrogenase from Leuconostoc mesenteroides (Sigma G5885).

Western blot and immunoprecipitation

Western blot

For Western blot procedures cardiac tissues were homogenized with RIPA buffer (ThermoScientific Pierce RIPA Buffer #89900) and protease inhibitors (Roche Cat. No. 11-836-170-001) using bullet blender at 4 °C for 10 minutes. Protein concentrations of supernatants were collected after centrifugation and quantified using Pierce™ BCA® Protein Assay Kits and Reagents (#23225). Protein lysates in Lammeli sample buffer were loaded to 10 % SDS-PAGE, transferred to PVDF membrane and blocked with 5% BSA in TSBT. Specific proteins were detected by specific antibodies listed above and corresponding secondary antibodies. Signals were visualized by HRP derived chemiluminescence (Amersham ECL Western Blotting Detection System #RPN2106) and film. Protein levels were quantified by Image-J.

Immunoprecipitation

In acetylation analysis with Acetyl-lysine specific immunoprecipitation (IP), 25 mg of human or murine cardiac tissue was homogenized in IP buffer (50mM Tris-Hcl pH 7.5, 10mM Sodium phosphate, 50mM Sodium Chloride, 0.1% Triton X-100, 1mM EDTA, 10µM Trichostatin A, 10mM nicotinamide, protease inhibitors. Homogenized tissue was cleared by 600 X g centrifugation for 10 minutes and supernatant incubated for 1hr on ice to release mitochondrial protein. The lysate was then incubated with Acetyl lysine antibody-conjugated to agarose (ImmuneChem, Burnaby ICP0388) or control immunoglobulin conjugated to agarose (Santa Cruz sc-2343) at 4 °C overnight with mild agitation. Agarose bound with acetylated proteins or immune controls were washed

gently with IP buffer followed by centrifugation to pellet beads. This wash step was repeated two times then two additional washes with cold PBS followed by centrifugation. Bound acetylated proteins were released with 0.1M glycine, pH 2.5 for 10 minutes at room temperature then pH to 7.0. SDS loading buffer was added and samples boiled for 5-minute at 95 °C. Samples were loaded to 10% SDS-PAGE gels for analysis.

Enrichment of CKM from myocardial tissue

CKM was extracted from pooled heart tissue (~200mg) in a two-step protocol involving Blue Sepharose CL6B affinity chromatography (Amersham Biosciences, HiTrap Blue HP GE17-0413-01) and cation-exchange chromatography Deae Sephadex™ A-50 Ion Exchange Chromatography Media, GE Healthcare 17-0180-01 as previously described in detail (2). Briefly, soluble protein from cytosolic fractions were obtained by homogenizing heart tissue with a dounce homogenizer followed by centrifugation steps to pellet nuclear and mitochondrial fractions. After initial homogenization suspension was spun at 600 X g for 10 minutes and pellet discarded. The supernatant was then spun 12,000 X g for 10 minutes to pellet mitochondria. The mitochondrial pellet was discarded and supernatant was spun again at 12,000 X g for 10 minutes. The supernatant (cytosolic fraction) were applied to a 5ml Blue Sepharose affinity column. The column was washed with 30ml of mobile phase (50mM sodium phosphate, pH 5.8) and protein eluted with 30ml of 50mM sodium phosphate (pH 8.5). Fractions containing CKM, as determined by activity and immunoblotting, were pooled, diluted in 50mM sodium phosphate (pH 5.8) and reapplied to the Blue Sepharose column. The column was washed 30ml of mobile phase (50mM sodium phosphate, pH 5.8) and CKM was

eluted with 30ml of 50mM sodium phosphate (pH 8.5). The elute was then applied to cation-exchange chromatography Deae Sephadex™ A-50 column on a linear salt gradient from 50mM to 480mM of NaCl. Peak CKM fractions were pooled, diluted in buffer then concentrated (Amicon) and saved for kinetic, immunoblot, and further experimental analysis.

Enrichment of MtCK from myocardial tissue

Extraction of MtCK from heart muscle was performed in a two-step protocol involving Blue Sepharose CL6B affinity chromatography (Amersham Biosciences, HiTrap Blue HP GE17-0413-01) and cation-exchange chromatography (Deae Sephadex™ A-50 Ion Exchange Chromatography Media, GE Healthcare 17-0180-01) adapted from (3). Briefly, the murine heart tissue was homogenized in buffer (220mM mannitol, 70mM sucrose, 10mM Hepes, 0.2mM EDTA, 1mM 2-mercaptoethanol (BME), 10µM Trichostatin A, 10mM nicotinamide, protease inhibitors, pH7. 4). The suspension was homogenized then centrifugation of the homogenate for 10 min at 600 X g. The supernatant was centrifuged for 20 min at 12,000 X g to pellet mitochondria. After washing the crude mitochondrial pellet with the above buffer the mitochondria were again pelleted with a centrifugation for 20 min at 12,000 X g. The enriched mitochondria fractions were pooled and then incubated overnight at 4 degrees in 50mM sodium phosphate, 1mM $MgCl_2$, 1mM BME, 0.2mM EGTA, 10µM Trichostatin A, 10mM nicotinamide, protease inhibitors, pH 8.0 under slow stirring. The lysate was centrifuged at 10,000 X g and resuspended and diluted in 50 mM sodium phosphate, 1 mM MgCl2, 1 mM BME, 0.2 mM EGTA, pH 5.8 and run on the on Blue-Sepharose column. Then the column was rinsed with 30ml of Blue-Sepharose buffer pH 5.8 and subsequently

with 30ml of the same buffer changed to pH 8.0. Then MtCK was eluted with the above buffer supplemented with 10mM ADP and the elute was run over cation exchange chromatography column on a linear salt gradient from 50 to 480mM of NaCl. Enriched MtCK fractions (between 190-240 mM NaCl) were pooled, concentrated, and used for downstream analysis.

Creatine kinase activity measurements and isozyme distributions.

Murine or human heart muscle (10 mg) was homogenized at 4°C in 50mM potassium phosphate buffer containing 1mM EDTA and 1mM b-mercaptoethanol, pH 7.4 (final concentration, 5 mg tissue/mL). Aliquots were removed for protein assays. Triton X-100 was then added to the homogenate at a final concentration of 0.1% and incubated at 1hr on ice. Total CK activity was measured in homogenates spectrophotometrically via a coupled-enzyme assay (4, 5). Using the same samples, individual CK isoenzymes were separated by electrophoretic mobility on an agarose gel and a coupled-enzyme assay performed as described (4). Absolute activities for each isoenzyme were calculated by multiplying relative isoenzyme activity (measured by densitometry) with total CK activity. Enzyme activities were measured (Units/mg protein).

Recombinant CKM, murine CKM, and murine MtCK activities were determined spectrophotometrically via coupled-enzyme assay as described previously (5). Hexokinase (Sigma) activity was determined by enzyme coupled assay using glucose-6-phosphate dehydrogenase.

Acetyl-CoA Concentration and NAD^+/NADH determination

Acetyl-CoA concentrations was determined in 10mg of cardiac tissue using the PicoProbe Acetyl CoA Assay Kit (Fluorometric) kit per manufacture instructions (Abcam ab87546). NAD^+ and NADH were determined in 8mg tissue using BioAssay Systems EnzyChrom NAD^+/NAD Assay Kit (E2ND-100) per manufacture instructions.

In Vitro Acetylation and Deacetylation Assays

For *in vitro* non-enzymatic acetylation reactions of murine CKM, MtCK, Hexokinase (Sigma), and human recombinant CKM (rCKM): Enzymes were incubated in acetylation buffer (50mM Hepes, 150mM NACl, pH 7.4) supplemented with the indicated concentrations of Acetyl-CoA (0µM, 25µM, 250µM, or 500µM) at time points of 15 minutes, 30 minutes, or 6 hours at 37 degrees with gentle rocking (400rpm). For enzymatic *in vitro* acetylation reactions the enzymes were incubated in the acetylation buffer supplemented with PCAF (Cayman Chemical Item No. 10009115) + 30µM Acetyl-CoA rocking (400rpm) at 37 degrees for 1 hour.

For *in vitro* deacetylation reactions enzymes were incubated in deacetylation buffer (50mM Tris-HCl, pH 7.5, 150mM NaCl, 1mM MgCl2, 0.5µM TSA) supplemented with Sirtuin 2 (Cayman Chemical Item No. 10011191) and NAD^+ concentrations of 0mM, 0.25mM, 0.5mM, or 1mM rocking (400rpm) at 37 degrees for 1 hour.

For downstream analysis after *in vitro* acetylation or deacetylaton reactions the reactions were either: (1) diluted 10-fold in appropriate enzyme activity assay buffer for specific activity measurements, (2) diluted in SDS Lammeli sample buffer and boiled 95 degrees for 5 minutes for immunoblotting, (3) diluted in Native-Page sample buffer and

run on native gels, (3) crosslinked per BDP cross link protocol below or liquid chromatography mass spectrometry analysis as described below.

Liquid chromatography mass spectrometry analysis of acetylated peptides and cross-linked peptides

Peptide samples were analyzed by liquid chromatography mass spectrometry using an Easy-nLC (Thermo Scientific) coupled to a Q Exactive Plus mass spectrometer (Thermo Scientific). Peptides were loaded onto a 3 cm x 100 μm inner diameter fused silica trap column packed with a stationary phase consisting of 5 μm Reprosil C8 particles with 120 Å pores (Dr. Maisch GmbH) with a flow rate of 2 μL/ min of mobile phase consisting of solvent A (H2O containing 0.1% formic acid) for 10 minutes. Peptides were then fractionated over a 60 cm x 75 μm inner diameter fused silica analytical column packed with 5 μm Reprosil C8 particles with 120 Å pores by applying a linear gradient from 95% solvent A, 5% solvent B to 60% solvent A, 40% solvent B over 120 minutes at a flow rate of 300 nL/min. Eluting peptide ions were ionized by electrospray ionization by applying a positive 2.2 kV potential to a laser pulled spray tip at the end of the analytical column. The mass spectrometer was operated using a top 20 data dependent acquisition method with a resolving power setting of 70,000 for MS1 and 17,500 for MS2 scans. Additional settings include an AGC target value of 1e6 with a maximum ion time of 100 ms for the MS1 scans and an AGC value of 5e4 with a maximum ion time of 100 ms for the MS2 scans. Charge state exclusion parameters were set to only allow ions with charge states from 2+ to 8+ to be selected for MS2. Ions selected for MS2 were isolated with a 3 m/z window and fragmented by HCD using a normalized collision

energy setting of 27. Ions for which MS2 was performed were then dynamically excluded from further selection for MS2 for 30 s.

Identification and Quantification of Acetylated Peptides and Proteins Raw MS data were processed using MaxQuant software (ver.1.6.17.0). Acetylated peptides were identified using the integrated Andromeda search algorithm (reference: Cox J, Neuhauser N, Michalski A, Scheltema RA, Olsen JV, Mann M (2011) Andromeda: a peptide search engine integrated into the MaxQuant environment. J Proteome Res 10: 1794–1805). Mouse samples were searched against Uniprot mouse protein sequence database (downloaded on April 28, 2018). Human samples were searched against Uniprot human protein sequence database (downloaded on April 28, 2018). Recombinant CKM data were searched against Uniprot Ecoli FASTA database (downloaded on August 6, 2019) spiked with rCKM sequence. The following parameters were used for searches: trypsin specificity with a maximum of 4 missed cleavages; carbamidomethylation of cysteine as fixed modification; oxidation of methionine, acetylation of protein N-term, and acetylation of lysine as variable modifications; instrument and MS/MS analyzer parameters used the default settings for Orbitrap; protein quantitation used only unmodified peptides and oxidized methionine; and all peptide and protein identifications filtered using 1% false discovery rate (FDR) cut-off from reversed sequence decoy database. Acetylated peptides were further filtered with localization probability score greater than 90%.

Cross-linked peptide samples were analyzed with the same LC-MS system described above using the same LC gradient conditions and mass spectrometer parameters with the following differences. The mass spectrometer was operated with a resolving power setting of 70,000 for MS1 and MS2 scans. Charge state exclusion parameters were set

to only allow ions with charge states from 4+ to 8+ to be selected for MS2. Ions selected for MS2 were isolated with a 3 m/z window and fragmented by HCD using a normalized collision energy setting of 30.

Salt Bridge Analysis

Salt bridge analysis was done using XLinkDB structure view and a 5 angstrom cutoff for detecting salt bridges.

Clustal Multiple Sequence Alignment

Multiple sequence alignment of KCRM across species was run using Clustal Omega tool (https://www.ebi.ac.uk/Tools/msa/clustalo/) (6).

Native Gels

Murine cardiac lysates or human recombinant CKM were run on 10% native-PAGE gels (1M Tris pH 8.8, 30%Bis-Acrylamide, dH_2O, 10% APS, TEMED). For cardiac lysates murine heart muscle (10 mg) was homogenized at 4°C in 50mM potassium phosphate buffer containing 1mM EDTA and 1mM b-mercaptoethanol, pH 7.4 (final concentration, 5 mg tissue/mL). Aliquots were removed for protein assays. Triton X-100 was then added to the homogenate at a final concentration of 0.1% and incubated at 1hr on ice. Tissue lysates or CKM were further diluted in Native-PAGE sample loading buffer (10% v/v glycerol, 0.0185% w/v Coomassie G-250) and then loaded into Native-PAGE gel without prior heating.

Native-PAGE running buffer (50mM Tris-Base, 50mM MOPS, 0.0375% SDS, pH 7.8) pre chilled to 4° C and kept at the temperature for the duration of the experiment.

Samples were then loaded into the gel and electrophoresis was conducted at constant voltage of 75V for 20 minutes followed by 100V for 1 hour at 4° C. Native gels were then transferred to PVDF membrane (100V for 1 hour at 4° C) and blocked with 5% BSA in TSBT. Dimeric versus monomeric CKM were detected by specific antibodies listed above and corresponding secondary antibodies. Reduced samples of CKM in SDS Lammeli sample buffer were also included as positive controls (as indicated in figure legends) on Native-PAGE gels to verify monomer band.

RNA Extraction and Quantitative Real-Time Polymerase Chain Reaction (RT-PCR)

Total RNA was isolated from frozen heart tissue using the RNeasy fibrous Kit (Qiagen). Omniscript reverse synthase and random hexamers were used for cDNA synthesis according to manufacturer's guidelines. Real Time PCR was performed using SYBR green (Bio-Rad). Results of mRNA levels were normalized to 18S rRNA levels and reported as fold-change over control. Primers used:

ANP: (Forward-*ATTGACAGGATTGGAGCCCAGAGT*) and (Reverse-*TGACACACCACAAGGGCTTAGGAT)*

BNP: (Forward-GCCAGTCTCCAGAGCAATTCA and (Reverse-GGGCCATTTCCTCCGACTT)

18S: (Forward-GGACAGGATTGACAGATTGATAG) and (Reverse-ATCGCTCCACCAACTAAGAA).

Statistics

All data presented as mean ± standard error of the mean (SEM). Statistical analysis was performed with GraphPad Prism 8.3 (GraphPad Software, San Diego, CA). Normal distribution of the data was analyzed using a Shapiro-Wilk test. Comparisons between 3 or more groups were conducted by one-way ANOVA followed by a Tukey post-hoc

analysis. For repeated measurements of multiple groups, 2-way repeated measure ANOVA was performed. Comparisons between 2 groups was done by unpaired, two-tailed t-test. All results were tested at the $P<0.05$ level of significance. For correlation graphs linear regression analysis was run on GraphPad Prism 8.3.

References

1. Karamanlidis G, Nascimben L, Couper GS, Shekar PS, del Monte F, and Tian R. Defective DNA replication impairs mitochondrial biogenesis in human failing hearts. *Circulation research.* 2010;106(9):1541-8.
2. Nuss JE, Amaning JK, Bailey CE, DeFord JH, Dimayuga VL, Rabek JP, et al. Oxidative modification and aggregation of creatine kinase from aged mouse skeletal muscle. *Aging.* 2009;1(6):557-72.
3. Schlegel J, Zurbriggen B, Wegmann G, Wyss M, Eppenberger HM, and Wallimann T. Native mitochondrial creatine kinase forms octameric structures. I. Isolation of two interconvertible mitochondrial creatine kinase forms, dimeric and octameric mitochondrial creatine kinase: characterization, localization, and structure-function relationships. *The Journal of biological chemistry.* 1988;263(32):16942-53.
4. Rosalki SB. An improved procedure for serum creatine phosphokinase determination. *The Journal of laboratory and clinical medicine.* 1967;69(4):696-705.
5. van Dorsten FA, Furter R, Bijkerk M, Wallimann T, and Nicolay K. The in vitro kinetics of mitochondrial and cytosolic creatine kinase determined by saturation transfer 31P-NMR. *Biochimica et biophysica acta.* 1996;1274(1-2):59-66.
6. Madeira F, Park YM, Lee J, Buso N, Gur T, Madhusoodanan N, et al. The EMBL-EBI search and sequence analysis tools APIs in 2019. *Nucleic Acids Res.* 2019;47(W1):W636-W41.

Figure S1: Contractile dysfunction, increased hypertrophy, and impaired creatine kinase system in mice after 12 weeks of chronic pressure overload. (A) Fractional Shortening (FS), **(B)** Cardiac hypertrophy (heart weight/body weight), and **(C)** Brain natriuretic peptide (BNP) and (ANP) mRNA values are reported as fold change from sham control mice, n=5 each group. **(D)** Densitometric quantification of CKM protein levels normalized to vinculin, n=5 per group. **(E)** Representative immunoblots of total protein and acetylation level of MtCK in failing hearts assessed by immunoprecipitation and Western blot (WB). **(F)** Densitometric quantification of MtCK protein level normalized to vinculin, n=5 for per group. **(G)** Correlation curve between the total CK activity level and CKM acetylation level in murine and human heart failure samples. A linear regression analysis was used, n=5 per group. **(H-J)** Correlation curve between the total CK activity level and MtCK acetylation level in murine and human heart failure samples. A linear regression analysis was used, n=5 per group. **(K)** Global protein acetylation were determined in TAC-stressed hearts by WB. Ponceau s staining was used as a loading control and CKM (bottom) as a sample processing control, n=5 for all groups. Data represents n=5 for all groups and shown as mean ± SEM. P value were calculated by one-way ANOVA followed by Tukey post-hoc analysis *P values <0.05, by Student t test **(A-C)**. Linear regression analysis for **G-J** was used.

Supplementary Table 1: clinical data of the human subjects. ICM: ischemic cardiomyopathy. DCM: dilated cardiomyopathy. BMI: body mass index. EF: ejection fraction.

Groups	Age	Gender	BMI	EF%	Diagnosis
Non-failing 1	58	Male	N/A	N/A	N/A
Non-failing 2	62	Female	N/A	N/A	N/A
Non-failing 3	60	Male	N/A	N/A	N/A
Non-failing 4	56	Male	N/A	N/A	N/A
Non-failing 5	50	Male	N/A	N/A	N/A
Failing 1	53	Male	28.4	15	DCM
Failing 2	60	Male	31.1	15	DCM
Failing 3	53	Female	22.1	15	ICM
Failing 4	59	Male	28.9	20	ICM
Failing 5	50	Male	26.9	15	ICM

Supplementary Table 2: creatine kinase isozyme distribution in myocardium

Groups	CKM%	MtCK%	MB%	BB%
Sham	63.2±1.9	33.0±2.0	3.16±0.41	0.64±0.12
TAC	56.8±1.6	38.1±1.6	4.24±0.06	0.80±0.04
Non-Failing	67.4±2.5	28.6±2.6	3.56±0.65	0.44±0.11
Failing	65.8±1.6	27.6±2.5	6.12±0.99	0.54±0.12

Supplementary Table 3: Identification and quantitation of lysine acetylation in enriched murine CKM with and without in vitro acetylation. The same acetylated lysine positions as identified in failing heart are indicated in red. Control (0μM) and treated (500μM) acetyl-CoA incubations.

Peptide	Position	Intensity treated	Intensity control
PFGNTHNK(Ac)FK	K9	3.18E+06	0.00E+00
FK(Ac)LNYKPQEEYPDLSK	K11	1.51E+07	0.00E+00
LNYK(Ac)PQEEYPDLSK	K15	3.48E+06	4.05E+06
VLTPDLYNK(Ac)LR	K41	6.85E+06	0.00E+00
HGGYK(Ac)PTDK	K101	8.28E+07	0.00E+00
HK(Ac)TDLNHENLK	K107	1.72E+07	0.00E+00
TDLNHENLK(Ac)GGDDLDPNYVLSSR	K116	8.90E+06	0.00E+00
AVEK(Ac)LSVEALNSLTGEFK	K156	6.64E+06	0.00E+00
LSVEALNSLTGEFK(Ac)GK	K170	4.85E+06	0.00E+00
K(Ac)AGHPFMWNEHLGYVLTCPSNLGTGLR	K266	1.94E+06	0.00E+00
GGVHVK(Ac)LANLSK	K298	2.04E+06	0.00E+00
VISMEK(Ac)GGNMK	K242	2.18E+06	0.00E+00
FCVGLQK(Ac)IEEIFKK	K259	9.61E+05	0.00E+00

Supplementary Table 4: Summary of all potential salt bridges between basic residues (R and K) and acidic residues (D and E). Red indicates residues on Chain A and Blue Chain B.

Chain 1	Residue 1	Atom 1	Chain 2	Residue 2	Atom 2	Distance (Å)
A	E46	OE1	B	K9	NZ	2.7
A	K177	NZ	B	D22	OD2	2.8
A	R148	NH2	B	D54	OD1	3.0
A	D22	OD2	B	K177	NZ	3.6
A	K196	NZ	B	D62	OD2	4.8
A	K45	NZ	A	D44	OD2	2.6
A	K223	NZ	A	D190	OD2	2.6
A	R152	NH2	A	D213	OD2	2.8
A	K41	NZ	A	D37	OD2	2.8
A	K32	NZ	A	D95	OD2	2.8
A	K11	NZ	A	D54	OD2	2.8
A	R151	NH1	A	D210	OD2	2.9
A	R148	NH1	A	D210	OD1	2.9
A	K101	NZ	A	D104	OD1	2.9
A	R314	NH1	A	E310	OE2	3.0
A	R292	NH2	A	D335	OD2	3.0
A	R252	NH2	A	E241	OE2	3.0
A	R251	NH2	A	E248	OE2	3.0
A	K365	NZ	A	D122	OD2	3.0
A	R316	NH2	A	D340	OD1	3.1
A	R251	NH2	A	E168	OE2	3.1
A	K307	NZ	A	D374	OD2	3.5
A	R252	NH1	A	E168	OE2	3.8
A	R209	NH1	A	D210	OD2	3.8
A	K319	NZ	A	E309	OE2	3.8
A	K86	NZ	A	D87	OD1	3.9
A	K366	NZ	A	D375	OD2	3.9
A	K265	NZ	A	E262	OE1	4.3
A	K86	NZ	A	D90	OD2	4.4
A	R236	NH1	A	E231	OE1	4.5
A	K156	NZ	A	E160	OE2	4.5
A	R252	NH1	A	E248	OE2	4.7
A	R236	NH1	A	E232	OE2	4.7
A	K381	NZ	A	D355	OD2	4.8
A	K247	NZ	A	D119	OD2	4.8
A	K358	NZ	A	E362	OE2	4.9

A	K105	NZ	A	E275	OE2	4.9
A	R151	NH1	A	D213	OD1	5.0
